# Extracellular electron transfer by the cultured coral photosymbiont *Symbiodinium microadriaticum*

**DOI:** 10.1007/s11120-026-01218-0

**Published:** 2026-05-25

**Authors:** Loris Marcel, James T. Simon, Joshua M. Lawrence, Svetlana Menkin, Adrian C. Barbrook, R. Ellen R. Nisbet, Christopher J. Howe, Jenny Z. Zhang

**Affiliations:** 1 Yusuf Hamied Department of Chemistry, University of Cambridge, Lensfield Road, Cambridge, CB1 2EW UK; 2 Department of Biochemistry, University of Cambridge, Tennis Court Road, Cambridge, CB2 1QW UK; 3 School of Biosciences, University of Nottingham, Sutton Bonington Campus, Sutton Bonington, LE12 5RD UK

**Keywords:** Extracellular electron transfer, Coral bleaching, Dinoflagellate algae, Photoelectrochemistry

## Abstract

Photosynthetic microorganisms may show extracellular electron transfer (EET), in which some of the electrons generated by photosynthesis or respiration are lost from the cell. Most studies have focussed on cyanobacteria, with very few on eukaryotic algae. Here we demonstrate EET from the eukaryotic dinoflagellate alga *Symbiodinium microadriaticum*, a species that can form symbiosis with corals and other Cnidaria. We show that the EET involves diffusible electroactive species, which may represent a previously unsuspected route for communication between symbionts and hosts. We show that EET can be used to study photosynthetic and respiratory functions in the dinoflagellate. We also show that it can provide information on the effects of environmental stresses including changes in temperature (linked to coral bleaching), pH and light intensity. The electrochemical platform outlined in this study offers a novel tool for studying dinoflagellate physiology, the coral-dinoflagellate symbiosis, and the molecular mechanisms of bleaching.

**Supplementary Information:**

The online version contains supplementary material available at 10.1007/s11120-026-01218-0.

## Introduction

The last fifteen years have seen a dramatic growth in our understanding of the ability of photosynthetic microorganisms to export electrons out of their cells in a bioelectricity-generating process termed extracellular electron transfer (EET) (Wey et al. 2019; Howe and Bombelli 2020). Although the role of EET in photosynthetic organisms is yet to be conclusively deciphered, it can provide experimental information on the bioenergetic processes of the organisms and/or subcellular fractions. EET may also be of interest as a sustainable source of modest amounts of electrical power (Wey et al. 2019; Lawrence et al. 2025; Howe and Bombelli 2023). Most studies on EET have been carried out with cyanobacteria. The EET rate has been shown to be influenced by both photosynthetic and respiratory electron transport activities and can provide information on the relative magnitudes of those activities in a cell (Wey et al. 2019; Saper et al. 2018; Kusama et al. 2022; Bombelli et al. 2011). Electrons can be generated by the action of Photosystem II or through respiration, although the exit point(s) from the photosynthetic and respiratory electron transport chains, and how the electrons actually leave the cell, are not fully clear. Cyanobacterial cells may secrete soluble mediators that assist in transferring electrons to the external environment or an electrode (Zhang et al. 2018; Wey et al. 2024). EET activity has also been studied in a small number of eukaryotic microalgae including diatoms (Laohavisit et al. 2015; Vicente-Garcia et al. 2024), *Chlamydomonas* (Anderson et al. 2016; Longatte et al. 2018), and the green alga *Parachlorella kessleri* MACC-38 (Petrova et al. 2024). There has been little use of EET to study the balance between respiratory and photosynthetic functions in eukaryotes, although crosstalk between the chloroplast and the mitochondrion is important in determining the overall energy balance of the cell (Shimakawa et al. 2024; Bailleul et al. 2015).

**Fig. 1:**
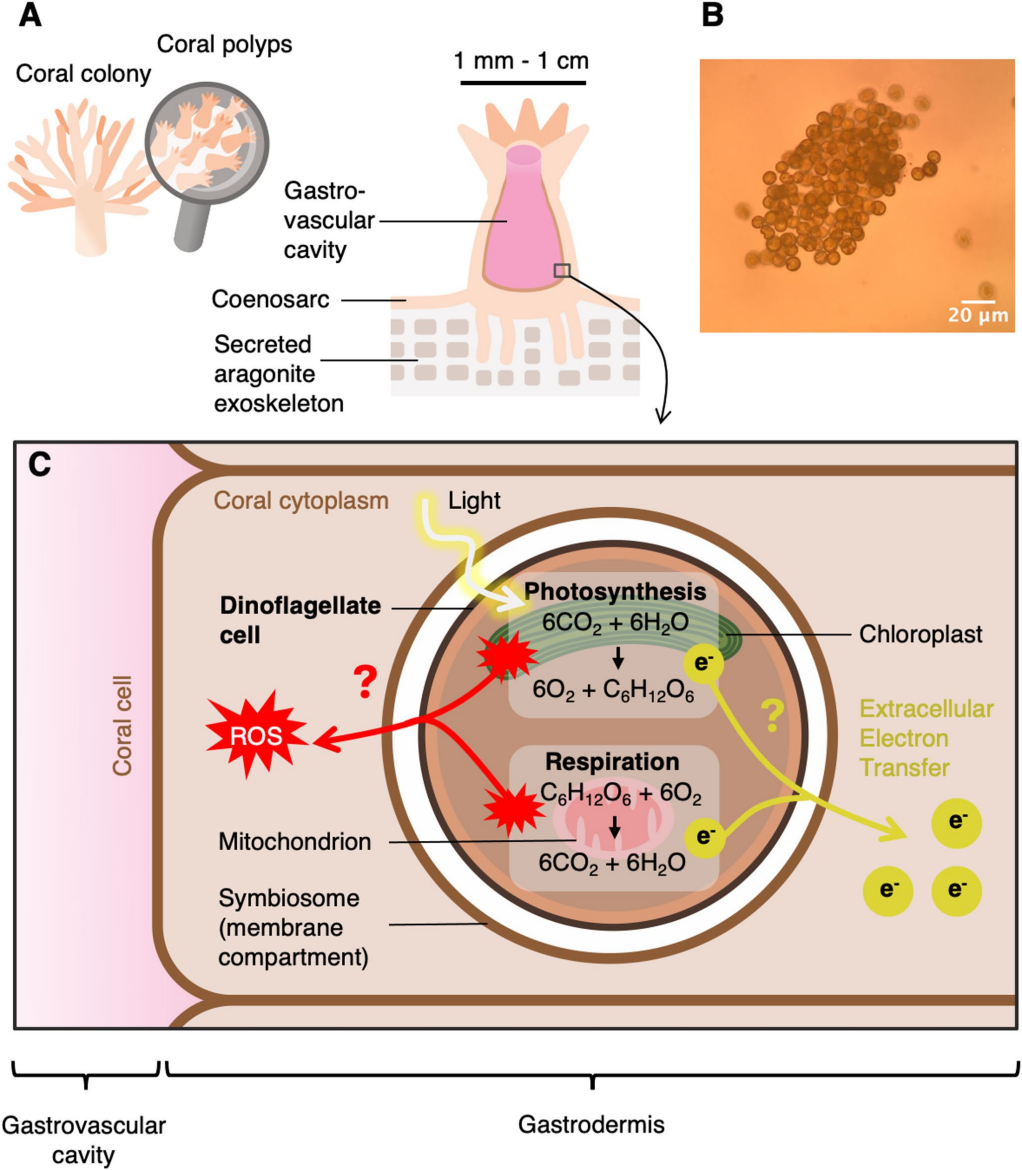
Interactions between corals and their dinoflagellate symbionts. (**A**) Anatomy of corals. A coral colony is composed of individual animals called polyps, which grow on a shared secreted aragonite exoskeleton and are connected by tissue (coenosarc). Polyps contain a large gastrovascular cavity, the lining of which is populated with symbiotic dinoflagellate algae together with other microorganisms (bacteria, eukaryotes, archaea and viruses). (**B**) Bright-field microscopy image of an aggregate of the model dinoflagellate studied, *Symbiodinium microadriaticum*. (**C**) Simplified schematic of the redox exchanges that could occur between dinoflagellates and their coral hosts. Dinoflagellate algae are maintained in the cytoplasm of coral cells, so their photosynthetic and respiratory pathways are likely to interact with the coral intracellular environment. Dinoflagellate cells used in this study were in grown in a free-living state only. Abbreviations: ROS, reactive oxygen species; e^−^, electrons

Our aim was to test EET activity in dinoflagellate algae and determine whether this could be used to study dinoflagellate physiology. Dinoflagellates are a widespread group of eukaryotic algae of great ecological importance (Gómez 2012). Around 50% of taxa are photosynthetic, with the group ancestrally having a red algal chloroplast acquired by secondary endosymbiosis and with the carotenoid peridinin as an accessory pigment. In a number of lineages, this peridinin-containing chloroplast has been lost or replaced (Dorrell and Howe 2015). Importantly, some dinoflagellates can form symbiotic relationships with a range of animals, including corals (Fig. 1A-B); they provide corals with sugars produced through oxygenic photosynthesis and carbon fixation in exchange for nitrogen, phosphate and inorganic carbon (Muscatine et al. 1989; Davy et al. 2012). In periods of environmental stress, such as increased water temperature, the coral-dinoflagellate symbiosis can break down (Helgoe et al. 2024). The dinoflagellates are subsequently lost from coral cells in a process called bleaching, which puts corals at risk of starvation. With climate change, coral bleaching events are becoming increasingly frequent, and corals may even become extinct by the end of the century, particularly in low-latitude regions (Mellin et al. 2024). Despite being essential members of reef ecosystems, studies of symbiotic dinoflagellates are hampered by, among other things, the difficulty of studying them *in hospite* and the poorly developed genetic tools (Chen et al. 2019; Gavin et al. 2026). Bleaching events are often correlated with algal redox stress (Szabó et al. 2020; Lesser 1996, 2024; Warner et al. 1999; Rosic et al. 2024; Rehman et al. 2016; Slavov et al. 2016), so information on the interplay between the photosynthetic and respiratory electron transport chains (Roberty and Plumier 2022), and the possibility of extracellular electron transfer to the host (Fig. 1C) could be useful in elucidating the molecular mechanisms of bleaching. Some studies of this interplay have been described (Pierangelini et al. 2020) but further work is needed and additional tools for studying dinoflagellate bioenergetics would be valuable.

Here, we develop an electrochemical platform to measure EET in the important symbiosis-forming peridinin-containing dinoflagellate species *Symbiodinium microadriaticum*. We show that *S. microadriaticum* performs EET, and provide evidence for the existence of soluble electron carriers. We demonstrate the dependence of EET on intracellular bioenergetics. This offers an instantaneous tool for studying dinoflagellate bioenergetics. We show that EET is responsive to changes in environmental conditions known to induce coral bleaching. We discuss the implications of dinoflagellate EET in the context of the coral-dinoflagellate symbiosis and for the development of coral diagnostics.

## Results

### Developing an electrochemical platform for analysing EET in *S. microadriaticum*

EET from photosynthetic microorganisms can be measured analytically in three-electrode electrochemical systems. These systems contain a working electrode, where the redox reaction of interest can be studied and compared against a well-characterised reference electrode. Typically, cells can be adhered to the working electrode to enable sensitive measurements of the biological current resulting from metabolic activity. In the case of photosynthetic cells, illumination causes an increase in this current, termed a photocurrent, which is caused by an enhancement of EET due to increased photosynthetic electron transfer activity (Wey et al. 2019; Saper et al. 2018). Measuring photocurrents provides a readout of photosynthetic activity as well as other pathways that may contribute to EET. Measuring changes in photocurrent as different potentials are applied between the working-electrode and a counter-electrode (stepped chronoamperometry) provides information on the redox species involved (Wey et al. 2019).

**Fig. 2:**
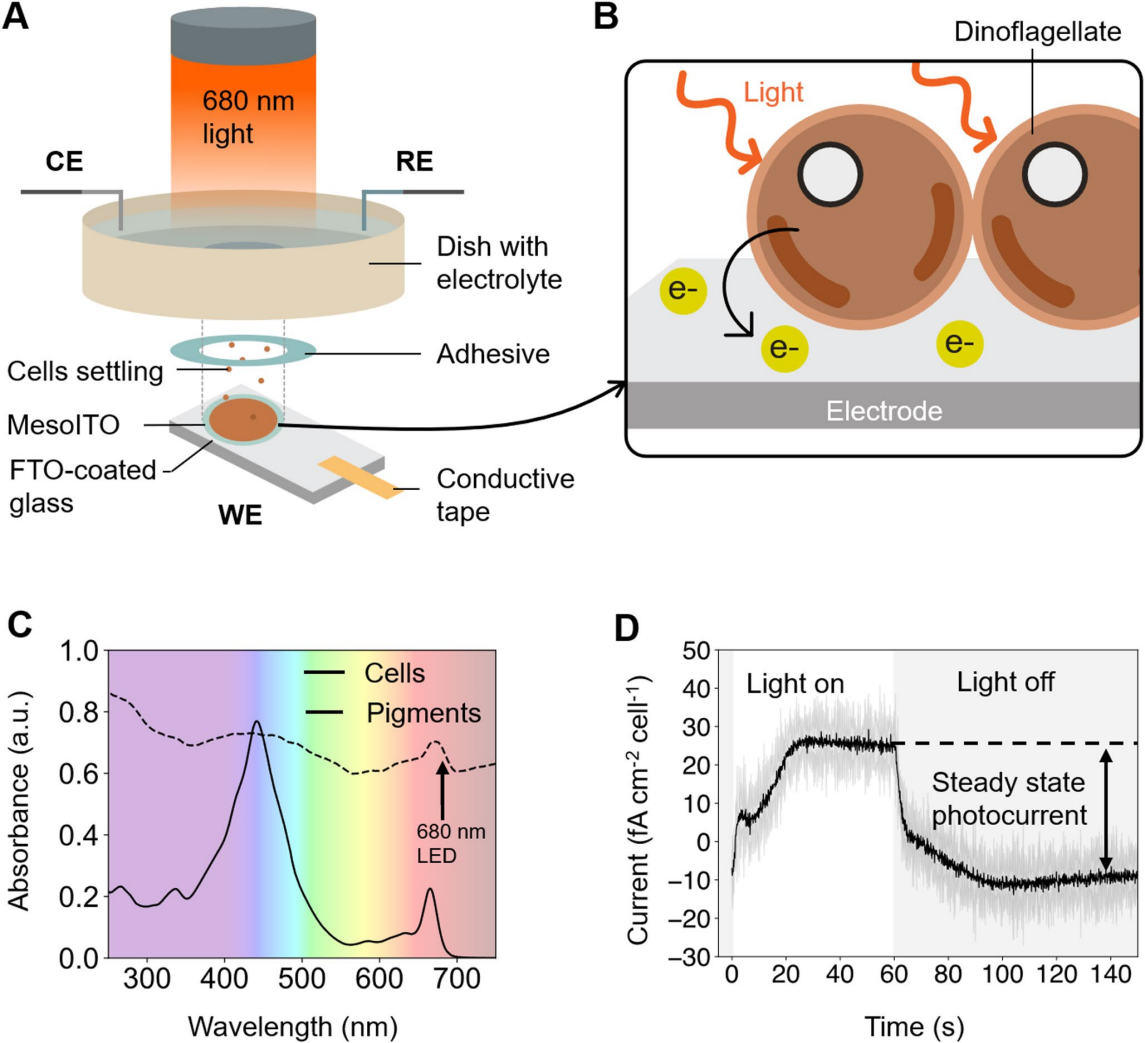
An electrochemical platform to study *S. microadriaticum*. (**A**) Schematic of the electrochemical platform used in this study. *S. microadriaticum* cells were settled onto a horizontal mesoITO electrode, which acted as the working electrode (WE) in a three-electrode system. An Ag/AgCl electrode and a platinum mesh were used as the reference and counter electrodes (CE and RE) respectively. Abbreviations: FTO, Fluorine-doped Tin Oxide. (**B**) Schematic of EET in dinoflagellates in which electrons leave the cell and reduce the electrode. (**C**) Absorbance spectrum of *S. microadriaticum* cells and their pigments extracted with methanol and ammonium acetate. (**D**) Photocurrent profile (current shape) obtained from *S. microadriaticum*. The steady state photocurrent is the magnitude of the current, defined by difference between the steady state currents in the light and in the dark. The photocurrent trace is the average of three biological replicates containing five technical replicates each, the shaded trace corresponds to standard deviation. Measurements were obtained at 0.3 V vs. standard hydrogen electrode (SHE); with 680 nm light at 50 µmol photons m^− 2^ s^− 1^; and 3 × 10^6^ cells

We designed a three-electrode system to measure EET in *S. microadriaticum*, a model species of symbiotic dinoflagellates (LaJeunesse 2017) (Fig. 2A). A horizontal working electrode was utilised (Fig. 2A-B, Supplementary Fig. 1A-B) because *S. microadriaticum* readily settles by forming aggregates (Fig. 1B). Mesoporous indium-tin oxide (mesoITO) was used as a working electrode due to its high surface area, biocompatibility and translucency (Kato et al. 2012). *S. microadriaticum* cells absorb strongly in red and blue light (Fig. 2C) due to the presence of chlorophylls and carotenoids involved in photosynthesis (Niedzwiedzki et al. 2014). A 680 nm light source was chosen to trigger photocurrents because ITO can produce abiotic currents in blue light (Brewer and Franzen 2004).

Using the three-electrode system, a photocurrent of ~ 25 fA cm^− 2^ cell^− 1^ was obtained (Fig. 2D) in the presence of *S. microadriaticum*. No photocurrent was detected in the absence of *S. microadriaticum* (Supplementary Fig. 1C). The large biotic current was unexpected, since *S. microadriaticum* has a thick cellulosic shell and a plasma membrane decorated by various glycans (Loeblich and Sherley 1979; Tortorelli et al. 2022) which could limit the diffusion of electron carriers out of the cell. The steady state photocurrent obtained with *S. microadriaticum* was 2.5-fold higher than reported for diatoms (which are also eukaryotic algae) without electron mediators (Vicente-Garcia et al. 2024) – although 680 nm light was used in our experiments instead of white light.

The bio-electrochemical system was further optimised by testing the effect of cell loading on photocurrent production (Supplementary Fig. 1D). Maximal steady state photocurrents were obtained between 3 and 6 × 10^6^ *S. microadriaticum* cells per 1 cm-diameter electrode (0.785 cm^2^). The steady state photocurrent was reached faster with 3 × 10^6^ cells per electrode than with larger numbers (Supplementary Fig. 1E), probably because larger cell numbers caused more shading of cells closest to the electrode. Hence this value was chosen for further experiments. Directly after pipetting *S. microadriaticum* onto the working electrode, the steady state photocurrent increased rapidly for 15 min, which was likely due to the cells settling onto the electrode (Supplementary Fig. 1F). After 15 min, photocurrents continued to increase slightly over time, possibly due to the acclimation of *S. microadriaticum* to light. Additionally, *S. microadriaticum* produced higher and more consistent currents when harvested from a stock culture with a concentration of around 1 × 10^6^ cells mL^− 1^ which was during period of steady growth (Supplementary Fig. 1G).

Measuring the steady state-photocurrent at a range of applied potentials (stepped chronoamperometry), we showed that the lowest potential where steady state photocurrents became positive was 0.2 V vs. the standard hydrogen electrode (SHE) (Supplementary Fig. 2A). Below 0.2 V vs. SHE, the reduction of photosynthetically-produced oxygen at the electrode produced large negative currents which may have hidden positive photocurrents, as shown by the cell-less control (Supplementary Fig. 2B) and the presence of photocurrent profile features at lower potentials (Supplementary Fig. 2C). Oxygen could not be removed, even with initial purging with N_2_ and enzymatic oxygen removal systems (Zhang et al. 2016), so it was not possible to make measurements at these low potentials without interference from oxygen reduction.

### EET in *S. microadriaticum* is mediated by endogenous diffusible redox molecules

**Fig. 3:**
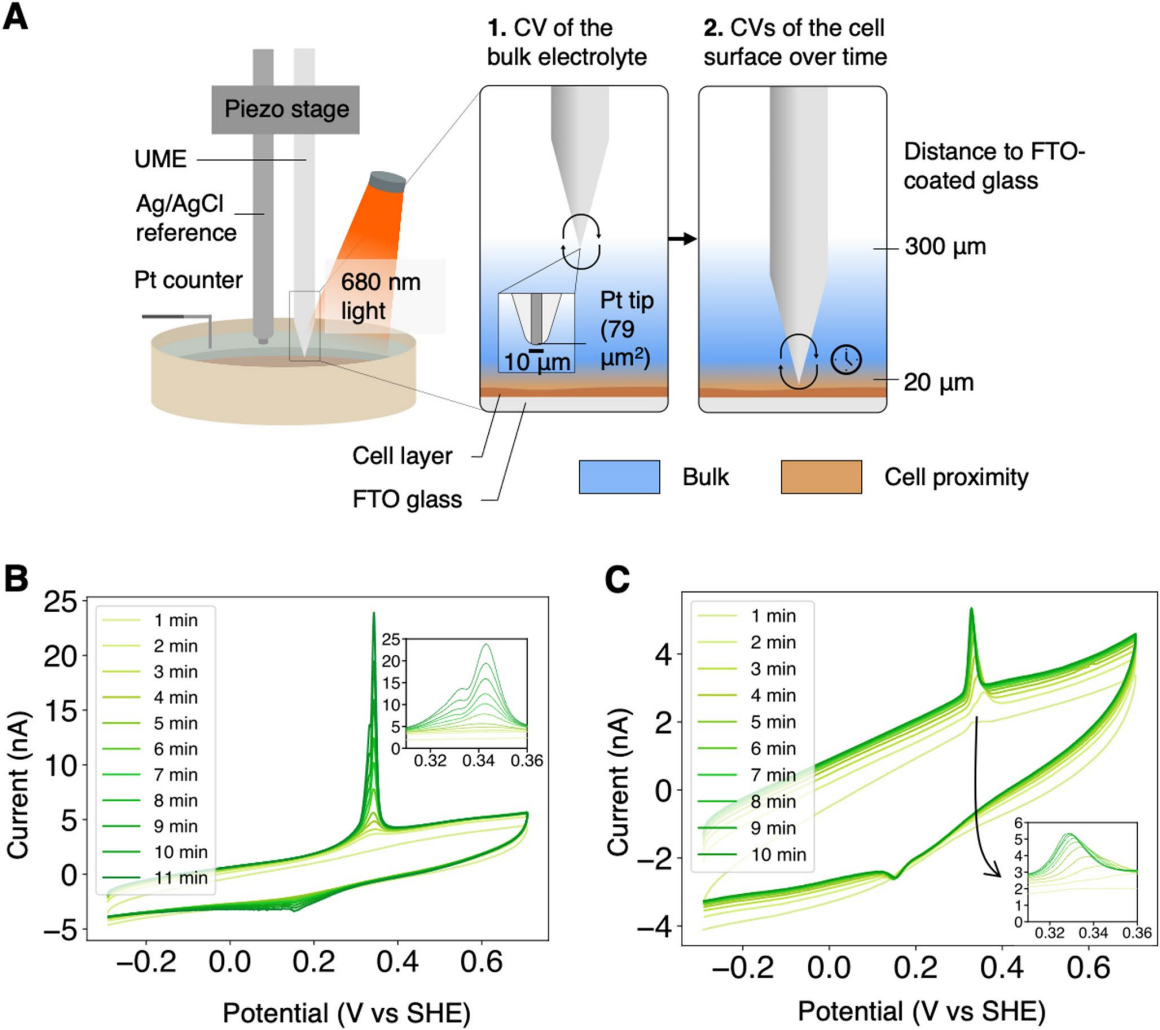
*S. microadriaticum* produces a diffusible electron carrier. (**A**) Schematic of the scanning electrochemical microscope (SECM) setup. The SECM is a three-electrode system where the working electrode is an ultra-microelectrode (UME) of diameter 10 μm. After two hours of incubation with or without 680 nm light at 100 µmol photons m^− 2^ s^− 1^, *S. microadriaticum* cells were subjected to (1) a cyclic voltammogram (CV) of the bulk, and (2) a CV of the cell surface. (**B**) Representative CVs of the cell surface after 2 h of incubation in light. (**C**) Representative CVs of the cell surface after 2 h of incubation in the dark. CV experiments were repeated 3 times. All CVs were performed at 50 mV s^− 1^ with a sample interval of 1 mV

In all electroactive organisms studied so far, electron export to the electrode was found to be either direct (via the direct contact of a protein or nanowire), or mediated by a diffusible species (Digel et al. 2024). To test which mode of electron transfer was involved for *S. microadriaticum*, an ultra-microelectrode (UME) operated by a scanning electrochemical microscope (SECM) platform was used. SECM platforms have been used previously to study redox activity from biological cells, including photosynthetic microorganisms (Saper et al. 2018) and plant thylakoids (McKelvey et al. 2013). Here, we made use of a three-electrode system in which the working electrode was a 10 μm-diameter platinum tip (Fig. 3A). The main advantage of SECM in the study of EET is that the working electrode can be precisely positioned in space using a piezo stage (Fig. 3A). This allows comparison of the redox activity both near and far from the dinoflagellates (Fig. 3A), and can therefore be used to investigate indirect EET mechanisms, unlike conventional three-electrode systems. Additionally, UMEs are very sensitive and can detect redox molecules in femto-molar concentrations (Chen et al. 2016).

The presence of redox species on the UME was assayed with cyclic voltammetry (CV). In CV, the potential at the working electrode is cycled, and redox species present on the electrode surface may be oxidised or reduced, resulting in a current. An abiotic control is presented in Supplementary Fig. 3A to show the background currents of the medium only. When *S. microadriaticum* cells were added to fresh medium, there was no redox response in the bulk electrolyte (Supplementary Fig. 3B) or at the cell surface (Supplementary Fig. 3C). After 2 h of incubation in either 100 µmol photons m^− 2^ s^− 1^ or darkness, oxidation peaks at around 0.33 V vs. SHE were detected in the bulk, suggesting that redox species were oxidised by the probe 300 μm away from the *S. microadriaticum* cells (Supplementary Fig. 3D). The oxidation peak detected was split into two peaks (Fig. 3B), suggesting that either multiple electron carriers were produced, or a main electron carrier underwent a sequential 2-electron oxidation process. The presence of the redox species in the dark is consistent with cells producing currents from respiratory activity, but the higher concentration of redox species after illumination shows that currents are mainly generated from photosynthetic activity. While the sharp peaks of the redox signals indicate that the electron carriers were adsorbed to the surface of the UME, the oxidation of the electron carrier in the bulk suggests that it diffused out of the *S. microadriaticum* cells to reach the UME probe. The observation that this peak is not present in the abiotic control (Supplementary Fig. 3A) suggests that the species originated from the cells themselves. Some of the redox activity could be contributed by metabolites produced by *S. microadriaticum*.

The spatial distribution of the detected redox species was then probed by performing CVs at the cell surface at regular time intervals. The concentration of the redox species increased as the measurements were performed, both in the light and the dark (Fig. 3B-C). Again, the sharp peaks of the redox signals implied that the electron carriers were gradually adsorbed onto the surface of the UME. Growth of the oxidation peak was greater in the presence of light, suggesting synthesis or secretion of these electron carriers was dependent on photosynthetic metabolism. This is supported by the complete inhibition of the oxidation peak in the presence of 100 µM 3-(3,4-dichlorophenyl)-1,1-dimethylurea (DCMU) (Supplementary Fig. 3E). DCMU is an inhibitor of photosystem II (PSII) which blocks plastoquinone reduction at the Q_B_ pocket of PSII, one of the first steps of the photosynthetic electron transfer chain (PETC). The fact that the electron carriers can be detected in the bulk, the dependence of their concentration on photosynthetic activity, as well as the fact their oxidation potentials (~ 0.33 V vs. SHE) are close to the potentials at which maximal photocurrents were observed in experiments with mesoITO (> 0.2 V vs. SHE, Supplementary Fig. 2A), all suggest that the electron carriers are responsible for EET in *S. microadriaticum*.

A reduction peak was also observed at 0.17 V vs. SHE, although this was notably smaller in area than the oxidation peak, and the separation between the reduction and oxidation peaks was larger than expected for a reversible redox process (Fig. 3B-C). This suggests the two peaks do not correspond to the same analyte, with each analyte undergoing irreversible oxidation or reduction at the surface of the UME. The analyte giving rise to the reduction peak is unlikely to contribute to the photocurrents collected when the applied potentials are more positive than 0.3 V vs. SHE. In contrast, the rate of EET is expected to be limited by the rate of synthesis of the analyte which gives rise to the oxidation peak. A control with no cells (without N_2_ purging) did not present any peaks after light incubation (Supplementary Fig. 3F), demonstrating the redox species giving rise to both the oxidation and reduction peaks were of biological origin and not due to degradation of f/2 medium components.

Overall, the results suggest that *S. microadriaticum* performs mediated EET. The electron carriers are diffusible, irreversibly oxidised by the electrode, and is produced in both light and dark albeit at different rates.

### EET provides information on intracellular bioenergetics of *S. microadriaticum*

In dinoflagellate algae, there are two main energy-producing pathways – photosynthesis and respiration – which are localised within the chloroplasts and mitochondria, respectively (Fig. 1C). To test whether EET would be a good reporter of intracellular bioenergetics in dinoflagellates, photocurrents were measured in the presence of inhibitors and substrates of these pathways.

**Fig. 4:**
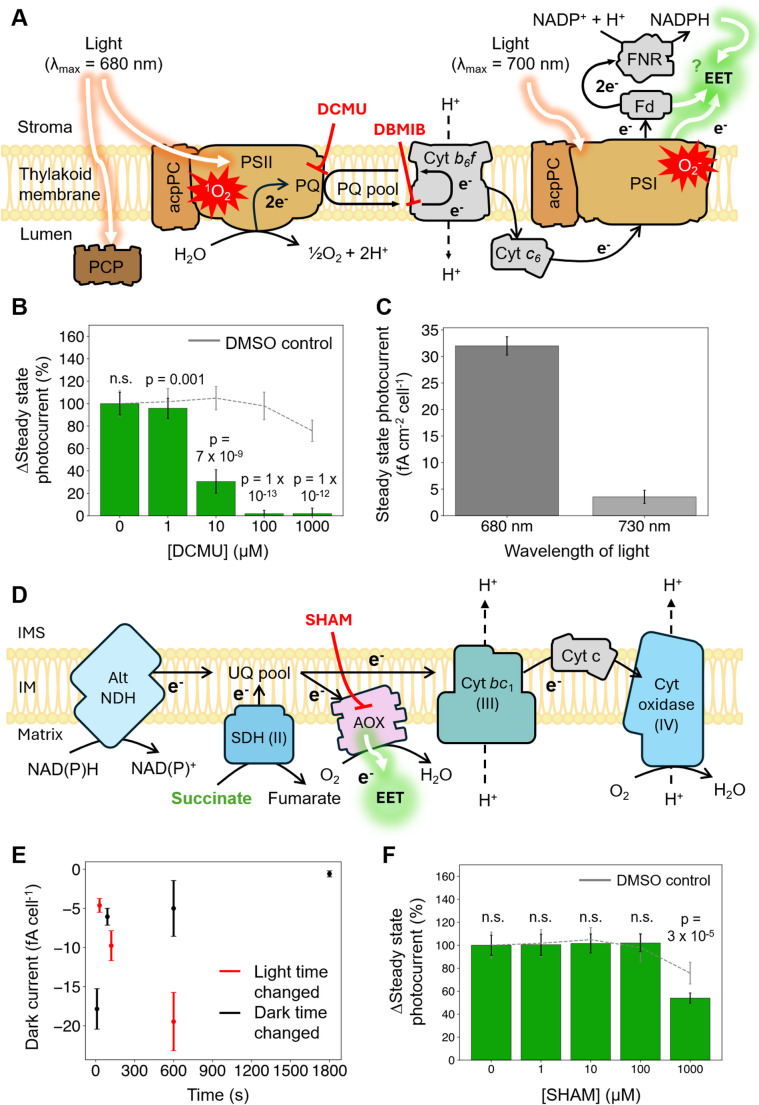
Extracellular electron export is dependent on intracellular bioenergetics. (**A**) The photosynthetic apparatus in *Symbiodinium* dinoflagellates. Abbreviations: PCP, Peridinin-chlorophyll *a* protein; acpPC, chlorophyll *a*-chlorophyll *c*_2_ peridinin protein complex; PQ, plastoquinone; Fd, ferredoxin; FNR Ferredoxin-NADP+ reductase; e^−^, electron; NADP+, nicotinamide adenine dinucleotide phosphate, ROS, reactive oxygen species, DCMU, 3-(3,4-dichlorophenyl)-1,1-dimethylurea; DBMIB, 2,5-dibromo-3-methyl-6-isopropylbenzoquinone. (**B**) Effect of DCMU on the steady state photocurrent versus a control with DMSO but no *S. microadriaticum*. (**C**) Effect of 680 nm and 730 nm light on the steady state photocurrent. (**D**) Schematic of the respiratory electron transport chain in *Symbiodinium* dinoflagellates. Abbreviations: Alt NDH, alternative NADH dehydrogenase; SDH, succinate dehydrogenase; AOX, alternative oxidase; SHAM, salicylhydroxamic acid; Cyt, cytochrome; IMS, intermembrane space; IM, inner membrane. (**E**) Steady state dark currents from light/dark cycles where either the light period was varied and the dark period was kept at 90 s, or where the dark period was varied and the light period was kept at 60 s. (**F**) Effect of SHAM on steady state photocurrent versus a control with DMSO but no *S. microadriaticum*. Data shown are averages of three biological replicates containing five technical replicates each, error bars represent the standard error of the mean. P-values were calculated with a paired samples t-test (*n* = 15). Unless specified, measurements were obtained at 0.3 V vs. SHE; with 680 nm light at 50 µmol photons m^− 2^ s^− 1^; and 3 × 10^6^ cells

First, photocurrents were measured under increasing conditions of DCMU which blocks plastoquinone reduction at the Q_B_ pocket of PSII (Fig. 4A). With increasing concentrations of DCMU, the steady state photocurrent decreased drastically, until it was fully abolished between 10 and 100 µM DCMU (Fig. 4B). Previously, it was shown that PSII activity could be inhibited in *Symbiodinium* sp. cells at DCMU concentrations ranging from 20 to 80 µM, both in free-living cells (Aihara et al. 2016) and in anemone hosts (Fransolet et al. 2014). This suggests that the steady state photocurrent is dependent on PSII activity. Additionally, steady state photocurrents were found to be 10-fold higher in 680 nm light – which activates both PSII and PSI – than in 730 nm light, which can activate PSI but not PSII (Zhao et al. 2024) (Fig. 4A, C). This shows that exported electrons may leave the PETC downstream of PSI but originate from water oxidation at PSII (Fig. 4A). To test whether electrons mainly exited the PETC at PSII or PSI, we added 2,5-dibromo-3-methyl-6-isopropylbenzoquinone (DBMIB) which inhibits plastoquinone oxidation at the cytochrome *b*_*6*_*f* complex. Addition of DBMIB abolished photocurrents at high concentrations (Supplementary Fig. 4A), suggesting that most of the electrons leave the PETC downstream of PSI (although at high concentrations, DBMIB can also reduce photosynthetic activity by quenching excited states of chlorophyll *a*). The inhibitory effect of DBMIB at lower concentrations is likely to be enhanced at higher light intensities (Vilyanen et al. 2024). An abiotic control with DBMIB did not produce photocurrents (Supplementary Fig. 4B).

Next, dark currents were measured in different light and dark incubation times. In the dark, photosynthetic electron transfer is inactive, so dinoflagellate algae obtain most of their reducing power from respiration which oxidises stored carbon-containing compounds to generate electrons. An increase in dark current would suggest that more electrons from respiration are being fed into the EET pathway. While higher respiratory rates are expected following longer light periods due to light-enhanced respiration (Falkowski et al. 1985), we observe the inverse in the dark current magnitude. Dark currents were more negative in longer light incubation times, with dark incubation times kept constant (Fig. 4E). It is unclear if this represents a dynamic regulation of EET activity in light transitions (e.g. due to thioredoxins (Hou et al. 2024)). A reductive current was observed from culture medium without *S. microadriaticum* (Supplementary Fig. 4C), indicating that redox species in the medium may have also contributed to the change in dark current.

Photocurrents were measured in the presence of salicylhydroxamic acid (SHAM, 0–1000 µM), an inhibitor of the mitochondrial alternative oxidase (AOX, Fig. 4D) which accounts for up to 26% of respiratory activity in dinoflagellate algae (Oakley et al. 2014). SHAM only showed an effect on EET from 1 mM (Fig. 4F), consistent with previous studies in which mM-range concentrations were needed to inhibit AOX-mediated respiration (Oakley et al. 2014). At this concentration SHAM caused a decrease, while an abiotic control did not produce any current (Supplementary Fig. 4D). It is possible that AOX itself might be a source of some of the respiration-derived electrons in EET, resulting in the decrease in the presence of SHAM. Addition of succinate, the substrate of complex II in the RETC (Fig. 4D), also induced a decrease in EET activity while an abiotic control did not produce any current (Supplementary Fig. 4E-F). The permeability of *S. microadriaticum* to succinate has not been shown before, but given the ability of other eukaryotic microalgae to take up and secrete succinate (Tomita et al. 2016; Meyer et al. 2022), it is likely that *S. microadriaticum* can import this metabolite. Since succinate is also a substrate in the tricarboxylic acid cycle, the decrease in steady state photocurrent could be explained by the activation of anabolic pathways at higher concentrations of succinate (Danne et al. 2012). This would draw reducing equivalents such as NAD(P)H into biosynthesis rather than respiration. Photocurrents were also measured in the presence of rotenone, an inhibitor of the canonical mitochondrial complex I. Complex I catalyses the first step of the RETC and oxidises NADH. Although it is not clear if the canonical complex I is present in dinoflagellate algae (and a type II NADPH-dehydrogenase is believed to be present) (Butterfield et al. 2013; Raven and Beardall 2017), rotenone slightly reduced the steady state photocurrent (Supplementary Fig. 4G), suggesting some effect on a canonical complex I or a possible inhibitory effect on other respiratory or photosynthetic proteins. Alternatively, because rotenone was poorly soluble and precipitated slightly, it may have caused a decrease in photocurrent through partial shading of *S. microadriaticum*. An abiotic control with rotenone produced no current (Supplementary Fig. 4H).

Together, these experiments suggest that EET and respiration are linked, with photosynthesis generating substrate molecules that are oxidised by the mitochondria and with some of the electrons subsequently fed into the EET pathway. This is consistent with previous reports of energetic coupling between photosynthesis and respiration found in *Symbiodinium* species (Pierangelini et al. 2020).

### EET activity is responsive to changes in environmental conditions linked to coral bleaching

To explore the use of the electrochemical platform for diagnostic applications, we measured photocurrents in different environmental conditions linked to bleaching. We hypothesised that the steady state photocurrent would vary in response to environmental changes.

**Fig. 5:**
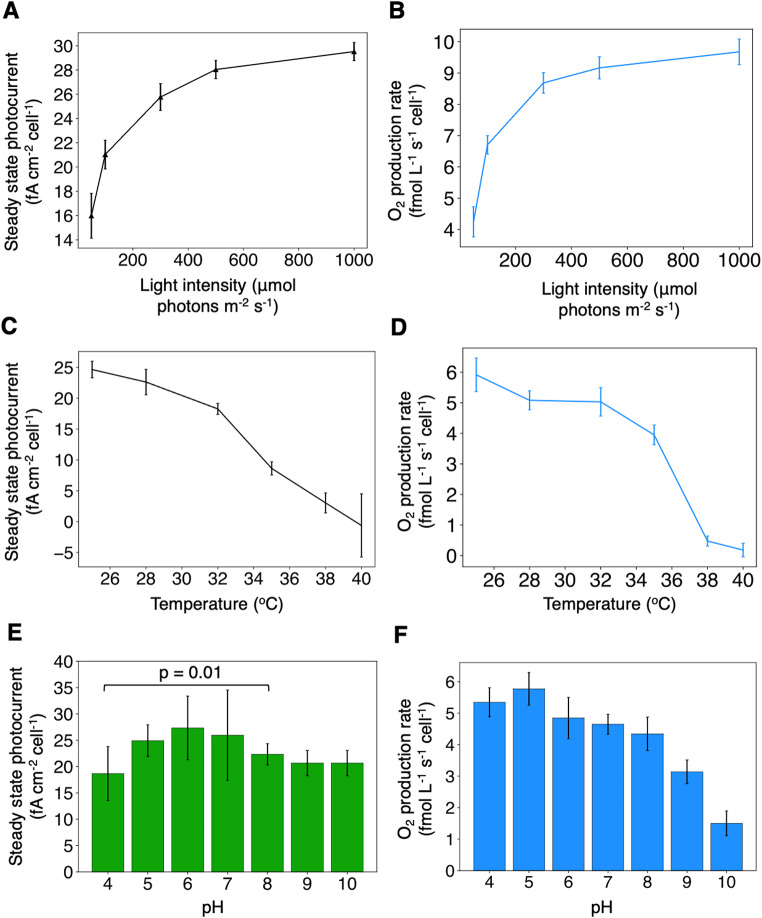
Extracellular electron export is responsive to changes in the environment. (**A**) Effect of light intensities ranging from 50 to 1000 µmol photons m^− 2^ s^− 1^ in increasing order after incubation in the dark for 1 h. (**B**) *S. microadriaticum* oxygen production levels over an increase in light intensity from 50 to 1000 µmol photons m^− 2^ s^− 1^. (**C**) Effect of short-term temperature change on steady state photocurrent. Temperature was kept constant for 15 min during each photocurrent measurements, then ramped up at 1.5 °C min^− 1^ to the next temperature. (**D**) Effect of short-term temperature change on oxygen production. (**E**) Effect of acute pH stress on steady state photocurrent. *S. microadriaticum* cells were centrifuged and resuspended directly from pH 7.8 to the measured pH. (**F**) Effect of acute pH stress on oxygen production rates. Data shown are averages of three biological replicates containing five technical replicates each, error bars represent standard error of the mean. Statistics were performed using unpaired samples t-tests (*n* = 15). Unless specified, measurements were obtained at 0.3 V vs. SHE; with 680 nm light at 50 µmol photons m^− 2^ s^− 1^; and 3 × 10^6^ cells

First, we tested the effect of light intensity. *S. microadriaticum* cells were kept in the dark for 1 h and were subsequently exposed to a series of light intensities. The light intensities ranged from 50 µmol photons m^− 2^ s^− 1^ (which represents typical growth conditions) to 1000 µmol photons m^− 2^ s^− 1^, which is higher than light intensities previously used to induce bleaching in corals (Jia et al. 2024) (and 680 nm light was used here instead of white light). Oxygen evolution was also measured using an oxygen sensor. The steady state photocurrent produced by *S. microadriaticum* increased with light intensity (Fig. 5A) whereas an abiotic control yielded no photocurrent (Supplementary Fig. 5A). A similar light-dependent increase was observed for oxygen production (Fig. 5B). EET activity was directly proportional to oxygen production in the light intensity range tested (Supplementary Fig. 5B), confirming the coupling between EET and photosynthesis.

Additionally, we measured photocurrents at different temperatures since increased water temperature is one of the main triggers of coral bleaching (Hughes et al. 2018; Helgoe et al. 2024). Steady state photocurrent density was strongly affected by temperature increase, decreasing on average by 4%/^o^C between 25 °C and 32 °C (Fig. 5C). In contrast, an abiotic control showed no photocurrent across the temperature range (Supplementary Fig. 6A). Growth rates of cell cultures also decreased with increasing temperatures (Supplementary Fig. 6B), suggesting a link between EET and cell health. However, the pH of the cell-less culture medium did decrease slightly at higher temperatures, which may have affected the cells (Supplementary Fig. 6C). Oxygen production rates decreased similarly with temperature (Fig. 5C), likely due to PSII damage and/or higher respiration rates. EET activity and oxygen production rates were positively correlated throughout the temperature range, though EET did not change fully linearly with respect to oxygen production (Supplementary Fig. 6D).

In the literature, pH stress was reported to affect coral calcification rates (Langdon and Atkinson 2005) but not to cause bleaching directly, except for one report (Anthony et al. 2008) – in that study, acidification was found to cause bleaching after 8 weeks of treatment, but alkalinisation was not tested. We measured photocurrents at different artificially adjusted pHs, hypothesising that there would be no strong effect on steady state photocurrent. Dinoflagellate cells were all grown at pH 7.8 and resuspended at the tested pH directly before EET measurement. NaOH and HCl were used to change the pH of the medium, which may not reflect natural conditions where pH is predominantly regulated by CO_2_ levels in the media, but can be used to explore a wider range of pH values. *S. microadriaticum* produced photocurrents at all the pHs tested although the steady state photocurrent was slightly lower at pH 4 (Fig. 5E). No photocurrent was obtained across the pH range in the abiotic control (Supplementary Fig. 7A). Although oxygen production was lower at alkaline pH values (Fig. 5F), EET remained stable. The lower oxygen levels were not due to solubility issues since pH did not affect oxygen solubility in the range tested (Supplementary Fig. 7B). EET activity and oxygen production did not show significant positive correlation in the case of pH change (Supplementary Fig. 7C). It is possible that the higher pH changed the midpoint potential of the redox species involved in EET, or facilitated the redox activity of components present in the electrolyte.

Overall, the results demonstrate that the electrochemical platform can give a snapshot of bioenergetics in environmental conditions linked to bleaching.

## Discussion

In this study, we developed an electrochemical platform to measure EET in *S. microadriaticum*. We confirm that EET activity can be measured and show that it depends on a diffusible extracellular mediator. Despite the low concentrations of electron carriers, the EET signal is detectable, stable and increases with light intensity - this is likely because the immobilisation of *S. microadriaticum* on the electrodes creates a confinement effect where the released redox molecules can accumulate without being lost to the bulk. We show that measurement of EET can be used as an instantaneous tool to probe intracellular bioenergetics, and that EET is responsive to stressors involved in coral bleaching. EET measurements can be used as a proxy for cell health, similar to the use described in the bioelectrochemical literature of EET measurements instead of growth rate because they provide better temporal resolution and reflect metabolic activity rather than optical density (Digel et al. 2024; Grobbler et al. 2018).

It will be particularly attractive in future studies to attempt to measure EET for cells inside the coral, although the contact between coral polyps and the electrode may need to be optimised using conductive polymers or a smaller electrode surface area. It is also possible that coral polyps also present EET activity, in which case EET signals from host and symbionts would need to be deconvoluted. Once adapted, our method would complement coral diagnostic methods monitoring photosynthetic activity with fluorimetry (Wangpraseurt et al. 2019; Beer et al. 1998). Fluorimetry is a powerful method for understanding whether the photosystems are in ‘open’ or ‘closed’ states, which indirectly give information about charge transfer along the PETC. However, its signals are tuned to observing non-photochemical quenching within PSII (less so PSI), can be complicated by quenching (for example by mediating quinone species (Rajagopal et al. 2003), and do not give any information on the final charges exiting the cells, as there are many deviating charge transfer processes that take place after photosystem I. As such our method has the advantage of being able to provide unique information about the bioenergetics of the cells, relating mostly to the terminal stages of charge transfer rather than the initial stages (which are measured by oximetry and fluorimetry). Our method could provide a readout of total electrons exported out of the cells, which would be an important factor when calculating the total redox balance under different conditions. This parameter has not been possible to measure until now without our method. These measurements would be particularly valuable, since many pathways other than photosynthesis can be involved in bleaching (Helgoe et al. 2024). The main limitation of this technique is the poor understanding of EET mechanisms in photosynthetic microorganisms. The photocurrent profile contains many information-rich transient peaks and troughs during light and dark stages. These likely arise from different dynamic processes with different kinetics occurring within the cells, as exemplified by the electrochemical study of a simpler photosynthetic thylakoid system (Lawrence et al. 2025). As such, this technique is still in the nascent stage and many of the signals still require assigning before it can be used to its full potential.

The observation of EET from dinoflagellate algae also opens up fundamental questions relating to the interactions between dinoflagellate algae and corals during symbiosis. Interspecies EET has been reported before in microbial communities (Wang and Sheng 2024; Nagarajan et al. 2013), and electromechanical communication between bacteria and eukaryotes has been demonstrated in engineered living materials (Atkinson 2024). However, to the best of our knowledge EET has not been reported between symbionts and their animal hosts. Although we have not demonstrated directly here that dinoflagellates in their symbiotic state can perform EET, we have shown that they do in their free-living state. Assuming they do so in their symbiont state as well (which seems likely, since they remain photosynthetically active), EET is likely to interact with the coral host cell. The possibility of EET-driven interaction is supported by the containment of symbionts within the symbiosome compartment ((Wakefield and Kempf 2001), Fig. 1C) and the diffusible nature of the electron carrier produced by *S. microadriaticum* (Fig. 4B-C). The symbiosome is decorated with transporters (Thies et al. 2022; Lin et al. 2015) that could transfer electron carriers across the membrane and into the coral cell.

There is little data in the literature on the metabolites exchanged between dinoflagellates and corals (often called ‘photosynthate’), let alone ones with redox activity. Therefore, this technique could complement metabolomics studies (Hillyer et al. 2016; Roach et al. 2021; Williams et al. 2021; Matthews et al. 2023), which have thus far focused mainly on carbohydrates and lipids (Rosset et al. 2021). Electrochemistry is commonly used to investigate the physical properties (both thermodynamics and kinetics) of catalysts, reaction substrates and products. Therefore, while the nature of the electron carrier in *S. microadriaticum* is still unknown, in the context of metabolomics, it could confirm whether redox active metabolites are released, estimate the quantity of the species involved, characterise their thermodynamic properties (directly measured by the mid-point potential), and any electro-reactivities they may exhibit either intrinsically or with other substances. Future studies will aim to characterise the electron carrier better. So far, the mediators for photosynthetic microorganisms have not been ascertained since the concentrations are typically low (McCormick et al. 2015), though in non-photosynthetic organisms there have been reports of phenazines, quinones to flavins mediating EET (Digel et al. 2024).

The fact that EET uses a diffusible mediator is particularly exciting, as it provides a mechanism for another level of interaction – the direct supply of reducing equivalents - between symbiont and host in addition to the other interactions that have been proposed. Direct supply of reducing equivalents could have several functions in the symbiosis. First, EET could be used to share energy with the coral host by means of reducing equivalents that funnel out excess energy from photosynthesis (Fig. 5A). This way, EET would benefit both corals and dinoflagellates, for which it could also act as a photoprotection pathway. Alternatively, EET may be involved in the initiation of bleaching by acting as a stress signalling pathway between dinoflagellate algae and coral hosts. For example, an increase in EET activity could be used by dinoflagellates to signal redox stress to their host before ROS-mediated damage can occur. Lastly, EET activity could be used to quench ROS. The electron carrier produced by *S. microadriaticum* could act as an electron donor to extracellular ROS, and prevent ROS from oxidising coral proteins instead. Future work will aim to explore the role of EET within the dinoflagellate-cnidarian symbiosis further.

Overall, we have demonstrated that the dinoflagellate *S. microadriaticum* performs EET. Assuming, as seems likely, this feature is common to the wider group of dinoflagellate algae, the electrochemical platform developed here will allow researchers to explore fundamental questions about the biology of these organisms. It will be particularly valuable in studying the effects of environmental stresses on dinoflagellates that can form symbioses with corals, broadening our understanding of the possible interaction pathways, and working towards building diagnostic tools for corals to mitigate the bleaching crisis.

## Methods

### Biological strains and culturing

Artificial seawater was prepared in MilliQ^®^ water by dissolving Coral Pro Salt (33.6 g L^− 1^, Red Sea) and tricine (0.5 g L^− 1^, $\:\ge\:$99%, 5704-04-1, Sigma-Aldrich), adjusting the pH to 7.8 with NaOH, and autoclaving. F/2 medium was made by adding a filter-sterilised f/2 supplement stock (0.15% v/v, Phytoplankton Nutrient Guillard’s F/2 Medium, Reef Phyto) to the artificial seawater medium. Wild-type *Symbiodinium microadriaticum* (strain CCMP2467) was cultured in liquid f/2 medium, without shaking, at 26 °C in a diel cycle of 14 h light and 10 h dark. The light intensity used was 20 µmol photons m^− 2^ s^− 1^ of photosynthetically active radiation. Cell counts were performed with a haemocytometer (Z359629-1EA, Merck). UV-vis spectrophotometry was performed with a Cary 60 instrument (Agilent Technologies, USA). Pigments were extracted with 0.5 M ammonium acetate and methanol as described previously (Rogers and Marcovich 2007).

### Three-electrode system fabrication

Working electrodes were made by cutting fluorine-doped tin oxide (FTO)-coated glass (735167-1EA, Sigma Aldrich) into 3 × 1.2 cm rectangles. To prepare the mesoITO substrate, the FTO-coated glass was cleaned by sonication at 37 kHz first in 99% isopropanol, then 99% ethanol. ITO nanoparticles (99.5%, 50926-11-9, ThermoFisher) were solubilised in acetic acid (5 M) in ethanol to produce a 20% w/v solution which was sonicated for 30 min on ice. Circular templates of 1 cm^2^ geometric area were prepared from adhesive tape and stuck onto the FTO-coated glass. The solubilised ITO was spread over the circular templates and scraped with a glass coverslip to obtain a thin layer of ITO (~ 2.5 μm). Once dried, the templates were removed and the electrodes were dried in a furnace (Carbolite ELF 11/14B/301) at 450 °C for 20 min, at a ramp rate of 4 °C min^− 1^. Platinum mesh counter electrodes were made by attaching 1 cm x 0.5 cm segments of platinum mesh (Goodfellow, UK) to tin-coated copper wires of diameter 1 mm. Ag/AgCl reference electrodes were made in-house and store in a saturated KCl solution. The reference electrodes were calibrated with a commercial Ag/AgCl electrode (MF-2052, BASi). To build the three-electrode setup, a custom-made polyether ether ketone dish with an opening at the bottom was fabricated.

### Chronoamperometry

Chronoamperometry was performed using a CompactStat.h potentiostat (Ivium Technologies). A three-electrode system composed of a working mesoITO electrode, an Ag/AgCl reference and a platinum mesh counter were used. The dish was filled with 5 mL f/2 electrolyte. To obtain 3 × 10^6^ *S. microadriaticum* cells, cell concentration was measured in the stock culture. Then, a volume containing 3 × 10^6^ cells was centrifuged at 3500 rpm for 3 min. The supernatant was removed and *S. microadriaticum* cells were resuspended in 100 µL fresh f/2 before being pipetted directly onto the electrode. Unless specified otherwise, measurements were performed at room temperature; with chopped 680 nm light (60 s light, 90 s dark) at 50 µmol photons m^− 2^ s^− 1^; 3 × 10^6^ cells; and a potential of 0.3 V vs SHE. Unless specified, chronoamperometry was performed at room temperature which may have varied between 20–25°C. A 730 nm LED (M730L5) was used to oxidise PSI in the chronoamperometry experiments. This LED was used with a bandpass filter (FB730-10 - Ø1”, 2.54 mm, Thorlabs) to block wavelengths lower than 720 nm. A pre-equilibration time of 90 s was used before each measurement. For stepped chronoamperometry, the potential was varied from 0.1 V to 0.6 V vs SHE. Where applicable, 3-(3,4-dichlorophenyl)-1,1-dimethylurea (DCMU, $\:\ge\:$98%, D2425, Merck), rotenone ($\:\ge\:$95%, 83-79-4, Sigma Aldrich), SHAM (99%, 11424533, Thermo Scientific) or 2,5-dibromo-3-methyl-6-isopropylbenzoquinone (DBMIB, 29096-93-3, Sigma Aldrich) were solubilised in DMSO (stocks of 100 mM), and added to the electrolyte.

### Scanning electrochemical microscopy

Scanning electrochemical microscopy was performed using a CH920C scanning electrochemical microscope (CH Instruments, USA). A three-electrode system composing a platinum wire (CH Instruments, USA), Ag/AgCl reference electrode (ALS, Japan) and a 10 μm diameter platinum UME with an RG value of 6 (CH Instruments, USA) were used. The UME probe was polished with 0.05 μm 𝛾-alumina prior to use. After 2 h of incubation in the light or in the dark, the following measurements were taken: a CV of the bulk 300 μm above the surface (50 mV s^−1^), and CVs 20 μm above the surface (50 mV s^−1^). Control CVs were performed at 10 mV s^−1^ with a sample interval of 1 mV. All experiments were performed at room temperature with, where applicable, 100 µmol photons m^−2^ s^−1^ of 680 nm light.

### Environmental change experiments

For light change experiments, *S. microadriaticum* cells were kept in the dark for 1 h and subsequently exposed to a series of increasing light intensities, ranging from 50 to 1000 µmol photons m^− 2^ s^− 1^. The light steps lasted 5 cycles of [60 s light + 90 s dark] each. For temperature change experiments, temperature was increased at a ramp rate of 0.5 °C min^− 1^ using a hot plate (for electrochemistry) or water bath (for oxygen evolution). At each measured temperature, the conditions were held for the duration of 5 cycles of [60 s light + 90 s dark]. For pH stress experiments, f/2 was adjusted to the desired pH using HCl or NaOH. All *S. microadriaticum* cells were grown at pH 7.8, centrifuged at 10,000 g for 3 min and resuspended directly into the measured pH, 10 min before the electrochemical measurement. EET measurements lasted 5 cycles of [60 s light + 90 s dark] each. All oxygen evolution rates were measured using an optical oxygen sensor (OXROB10-HS, Pyroscience) connected to a multi-analyte meter (FSPRO-2, Pyroscience). An upper calibration of the oxygen sensor was performed in air-saturated water as per the manufacturer’s instructions. Temperature was measured when calibrating to account for changes in oxygen solubility (a dipping probe (Pt100, Pyroscience) was used).

### Data analysis

Steady state current intensities I_light_ and I_dark_ were calculated by averaging the last 5s of the current in the light and dark respectively. Steady state photocurrent was calculated by subtracting I_dark_ from I_light_. Potentials measured vs. Ag/AgCl were converted to potentials vs. SHE with the following conversion method: E_SHE_ = E_Ag/AgCl_ + 0.209 V. All the data analysis was performed using in-house Python scripts, which can be found on GitHub (https://github.com/Zhang-Lab-Cambridge/DinoBot.git). Paired and unpaired t-tests were performed in Python using the SciPy library. T-tests were performed over 3 biological replicates containing 5 technical replicates each. The level of significance was chosen as 0.05. Linear regression analysis was carried out in python using the statsmodel module.

## Electronic Supplementary Material

Below is the link to the electronic supplementary material.

Supplementary Material 1

## Data Availability

All data is available on GitHub at https://github.com/Zhang-Lab-Cambridge/Bioelectricity-generation-by-Symbiodinium-microadriaticum.git.
