# Supplementary material for: Extracellular electron transfer by the cultured coral photosymbiont Symbiodinium microadriaticum
Source: Photosynth Res. 2026 May 25;164(3):31. doi: 10.1007/s11120-026-01218-0 (PMC13201290; doi:10.1007/s11120-026-01218-0)
Supplement: Supplementary file 1 — Supplementary Material 1 [file 11120_2026_1218_MOESM1_ESM.docx]

**Extracellular electron transport by the cultured coral photosymbiont *Symbiodinium microadriaticum***

**Photosynthesis Research**

Loris Marcel^1,2^, James T. Simon^1^, Joshua M. Lawrence^1,2^, Svetlana Menkin^1^, Adrian C. Barbrook^2^, R. Ellen R. Nisbet^3^*, Christopher J. Howe^2^*, Jenny Z. Zhang^1^*

^1^Yusuf Hamied Department of Chemistry, University of Cambridge, Lensfield Road, Cambridge, CB1 2EW, UK

^2^Department of Biochemistry, University of Cambridge, Tennis Court Road, Cambridge, CB2 1QW, UK

^3^School of Bioscience, University of Nottingham, Sutton Bonington Campus, Sutton Bonington, LE12 5RD, UK

Corresponding authors email:

Jenny Z. Zhang: [jz366@cam.ac.uk](mailto:jz366@cam.ac.uk)

Christopher J. Howe: [ch26@cam.ac.uk](mailto:ch26@cam.ac.uk)

R. Ellen R. Nisbet: [Ellen.Nisbet@nottingham.ac.uk](mailto:Ellen.Nisbet@nottingham.ac.uk)

|  |  |
| --- | --- |
| Fig. S1: Optimization of the electrochemical platform | 2 |
| Fig. S2: Stepped chronoamperometry of *S. microadriaticum* cells on mesoITO | 4 |
| Fig. S3: Controls for SECM experiments | 6 |
| Fig. S4: Effect of respiratory activity on EET in *S. microadriaticum* | 7 |
| Fig. S5: Response of *S. microadriaticum* cells to different light intensities | 9 |
| Fig. S6: Response of *S. microadriaticum* cells to different temperatures | 10 |
| Fig. S7: Response of *S. microadriaticum* cells to different pH levels | 11 |


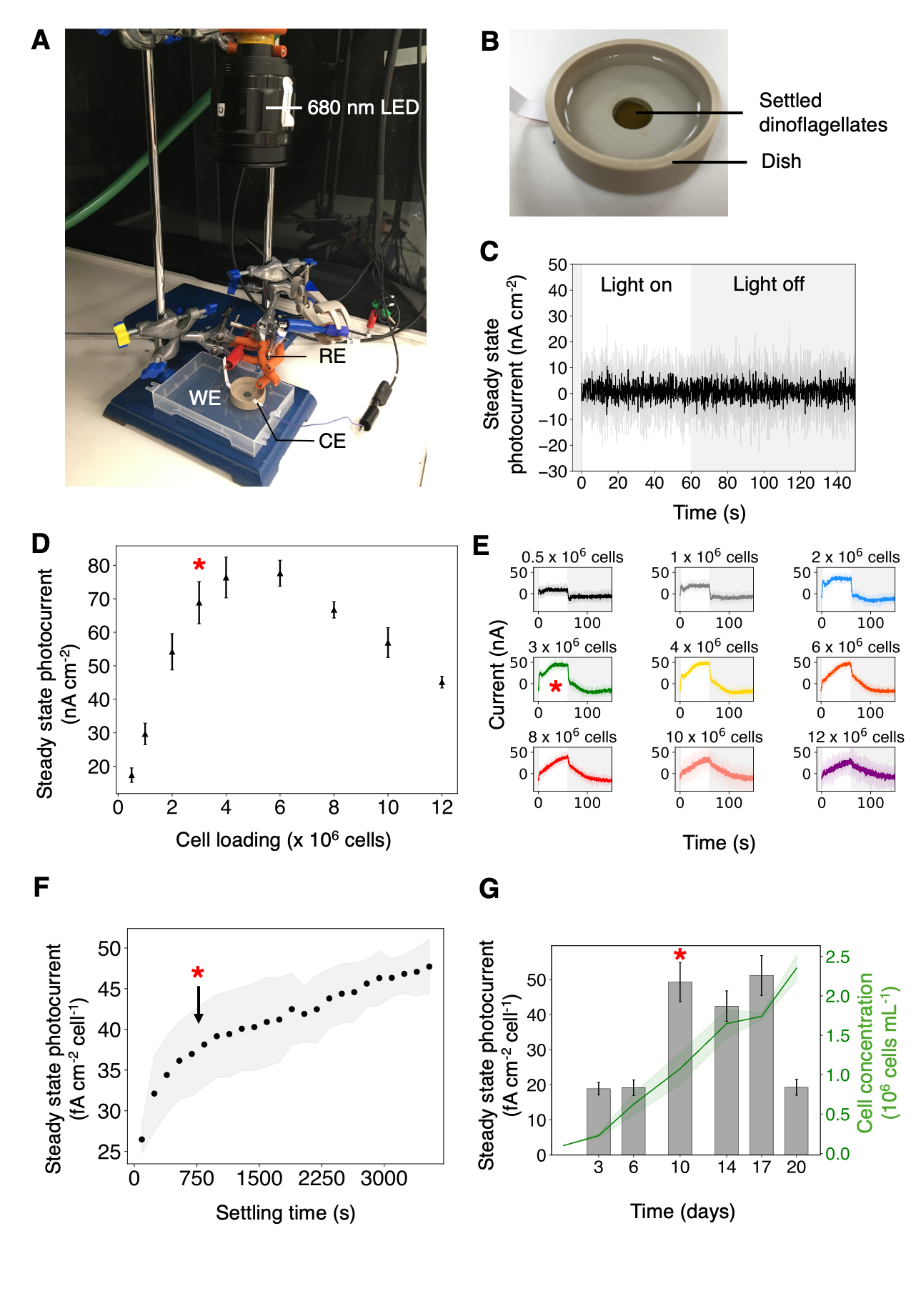


Fig. S1.

Optimization of the electrochemical platform**.** (**A**) Image of the electrochemical platform. (**B**) Electrochemical dish with *S. microadriaticum* cells settled onto the working electrode. (**C**) Photocurrent trace on mesoITO without cells. (**D**) Effect of cell loading on steady state photocurrent. (**E**) Effect of cell loading on photocurrent profile. (**F**) Steady state photocurrent over time as *S. microadriaticum* cells settle onto the working electrode. T_0_ corresponds to the time the cells were added into the electrochemical dish. The initial period of rapid increase may represent settling, which is complete by 15 min. (**G**) Effect of growth stage on steady state photocurrent. Data shown are averages of three biological replicates containing five technical replicates each, error bars and shaded areas represent the standard error of the mean. Unless specified, measurements were obtained at 0.3 V vs SHE; with 680 nm light at 50 μmol photons m^-2^ s^-1^; and 3 x 10^6^ cells. Symbols (*) represent optimized conditions.


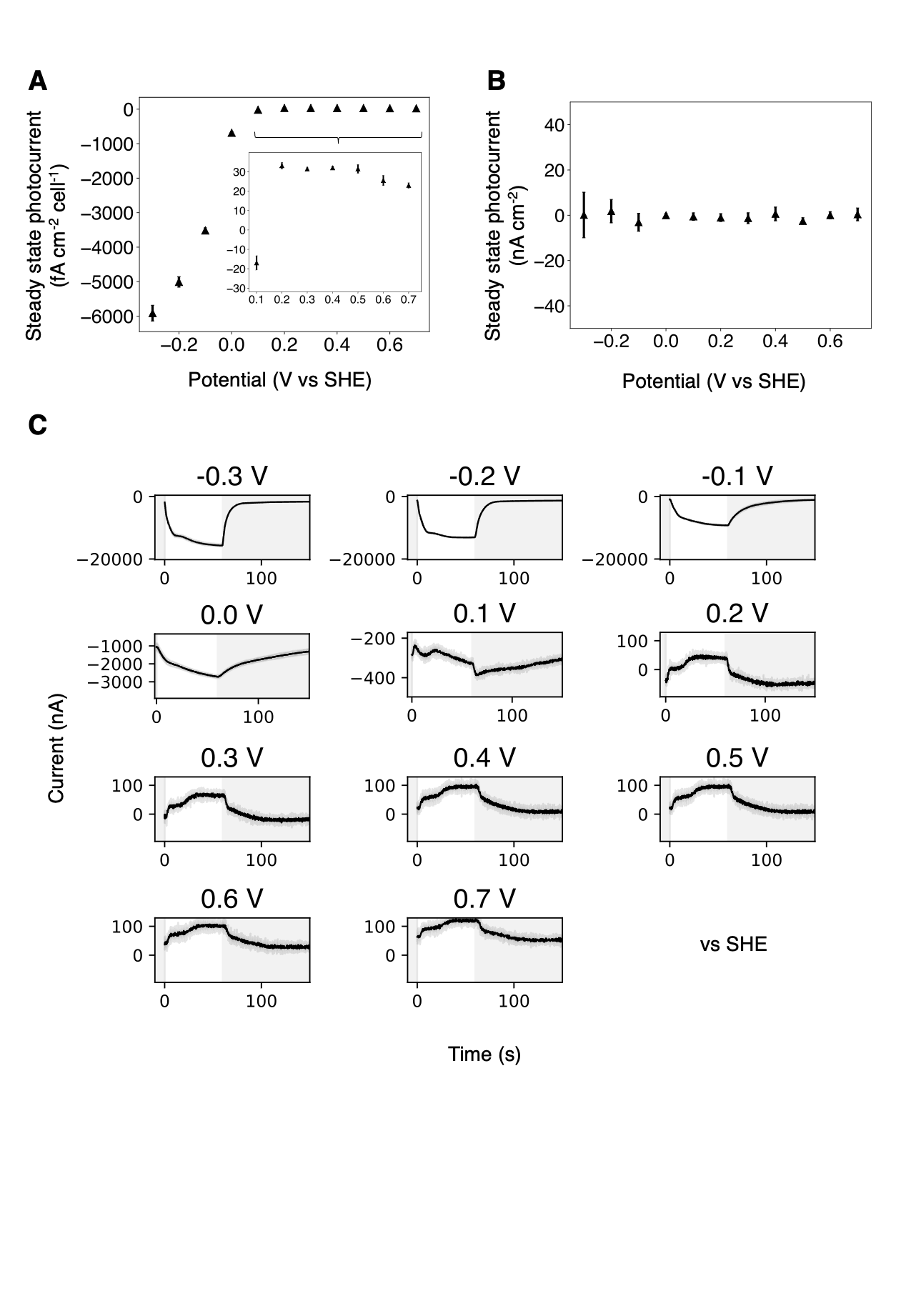


Fig. S2.

Stepped chronoamperometry of *S. microadriaticum* cells on mesoITO. (**A**) Steady state photocurrents obtained during stepped chronoamperometry from –0.3 V to 0.7 V vs SHE with *S. microadriaticum* added. Inset: steady state photocurrents in the 0.1 V to 0.7 V range. (**B**) Steady state photocurrents obtained during stepped chronoamperometry from –0.3 V to 0.7 V vs SHE, without *S. microadriaticum* added. (**C**) Photocurrent profiles obtained from the stepped chronoamperometry. Data shown are averages of three biological replicates containing five technical replicates each, error bars represent the standard error of the mean and shaded areas represent standard deviation. Measurements were obtained with 680 nm light at 50 μmol photons m^-2^ s^-1^ and 3 x 10^6^ cells.


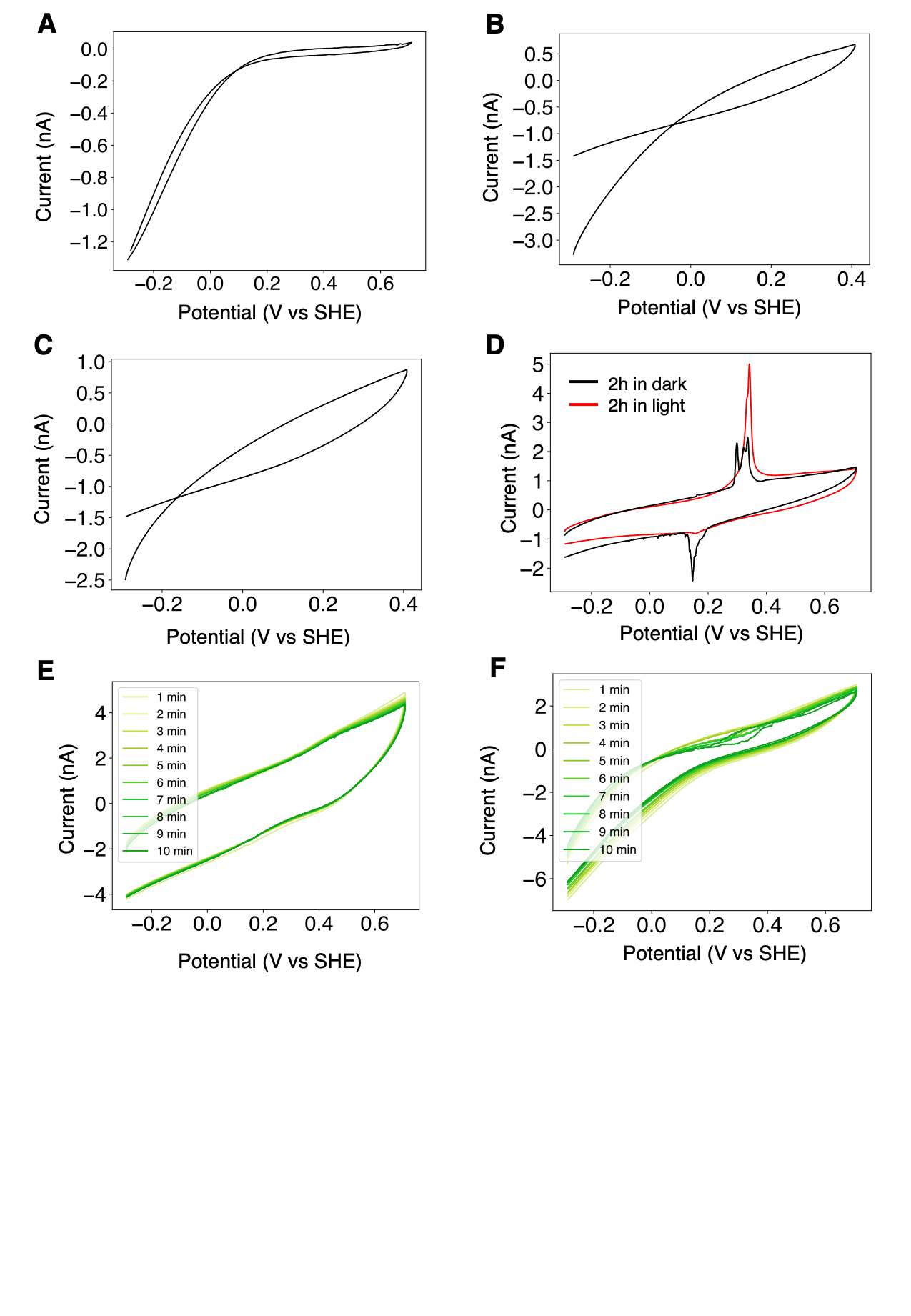


Fig. S3.

Controls for SECM experiments**.** (**A**) CV of f/2 medium. (**B**) Bulk CV of fresh f/2 medium to which *S. microadriaticum* cells were added. (**C**) CV of fresh f/2 medium to which *S. microadriaticum* cells were added, at the cell surface. (**D**) Average CVs of the bulk after 2h of incubation, in light or dark (n = 3). (**E**) Consecutive CVs of the surface with cells after 2 h of illumination and 100 μM DCMU. (**F**) Consecutive CVs of the surface without cells after 2 h of illumination. B-C and were performed at 10 mV s^-1^, the other CVs were performed at 50 mV s^-1^ and a sample interval of 1 mV. Electrolyte was not purged with N_2_.


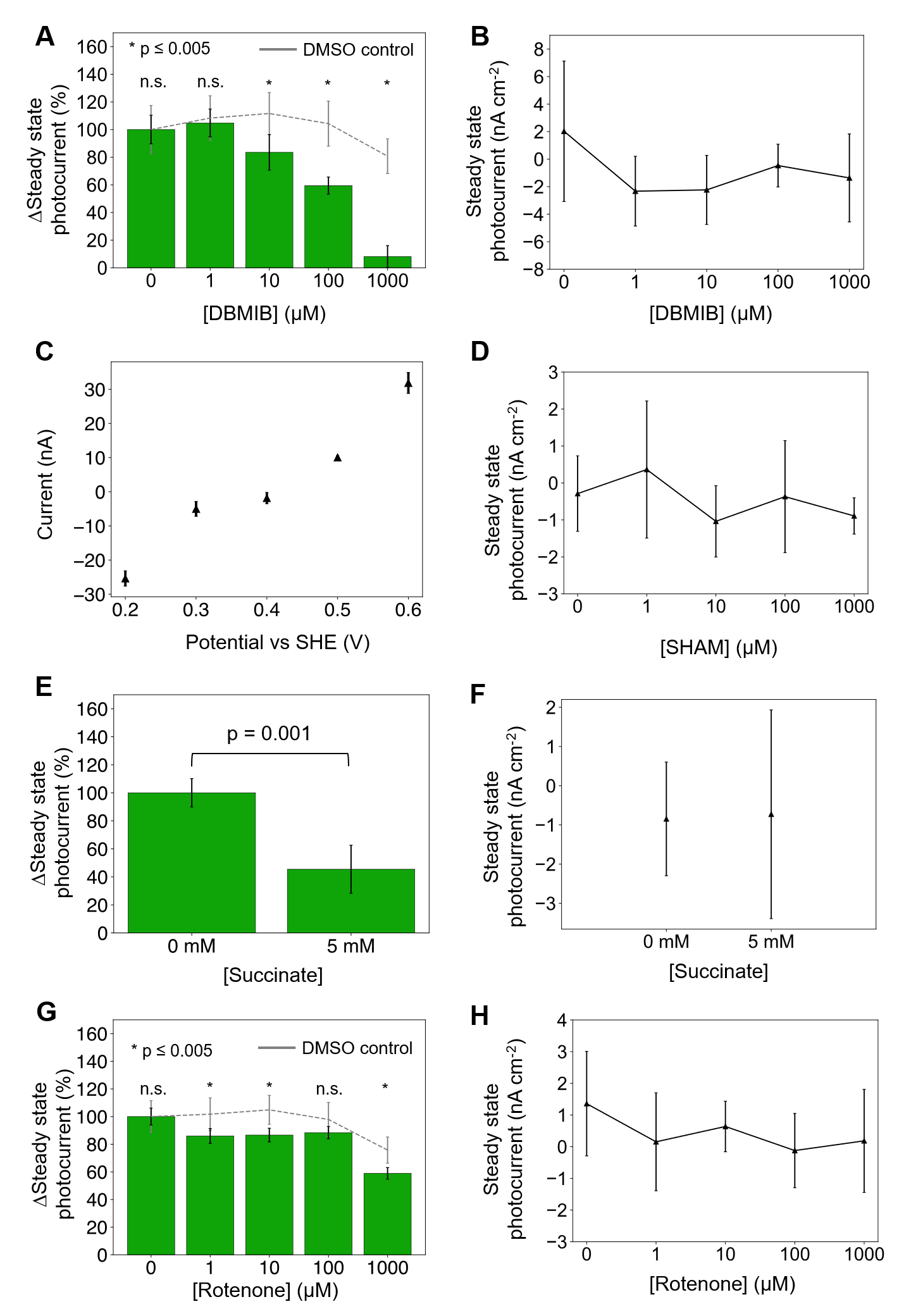


Fig. S4.

Effect of respiratory activity on EET in *S. microadriaticum*. (**A**) Effect of DBMIB on the steady state photocurrent versus a control with DMSO but no *S. microadriaticum*. (**B**) Abiotic control with DBMIB. (**C**) Dark currents obtained during stepped chronoamperometry from 0.2 V to 0.6 V vs SHE in f/2 medium without cells. (**D**) Abiotic control with SHAM. (**E**) Effect of succinate on the steady state photocurrent. (**F**) Abiotic control with succinate. (**G**) Effect of rotenone on the steady state photocurrent versus a control with DMSO but no *S. microadriaticum*. (**H**) Abiotic control with rotenone. Data shown in A, B and G are averages of three biological replicates containing three technical replicates each. Data shown in B and C are averages of three biological replicates containing five technical replicates each. Data shown in the other panels are averages of five technical replicates each, error bars represent standard deviation. Statistics were performed using an unpaired samples t-test for A and G, and a paired samples t-test for E (n = 15). Unless specified, measurements were obtained at 0.3 V vs SHE; with 680 nm light at 50 μmol photons m^-2^ s^-1^; and 3 x 10^6^ cells.

**
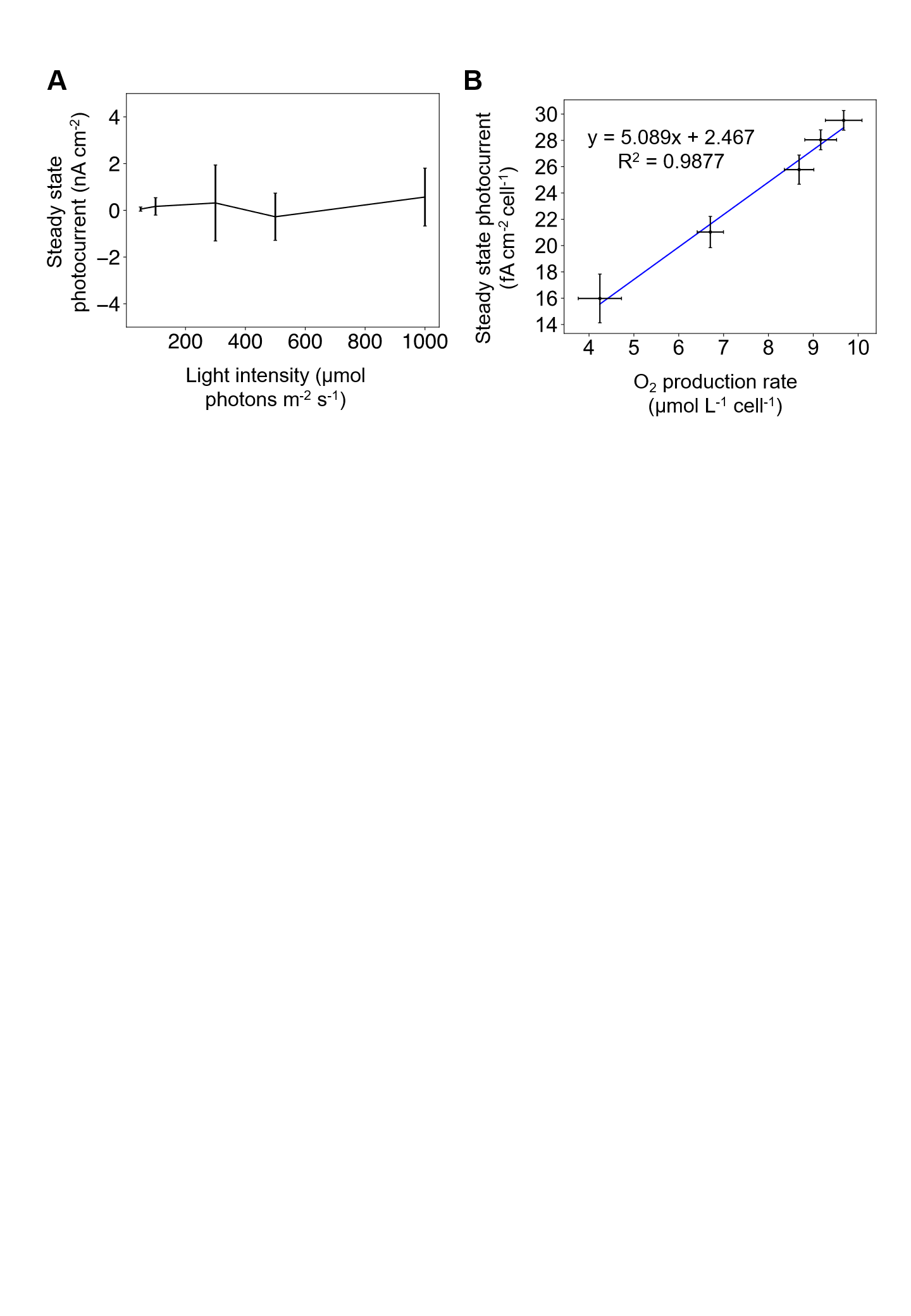
**

Fig. S5.

Response of *S. microadriaticum* cells to different light intensities. (**A**) Steady state photocurrents at increasing light intensities without *S. microadriaticum* added. (**B**) Linear regression analysis of steady state photocurrent versus oxygen production in increasing light intensity. For B, data shown are averages of three biological replicates, error bars represent the standard error of the mean. Other data shown are averages of five technical replicates, error bars represent standard error of the mean.

**
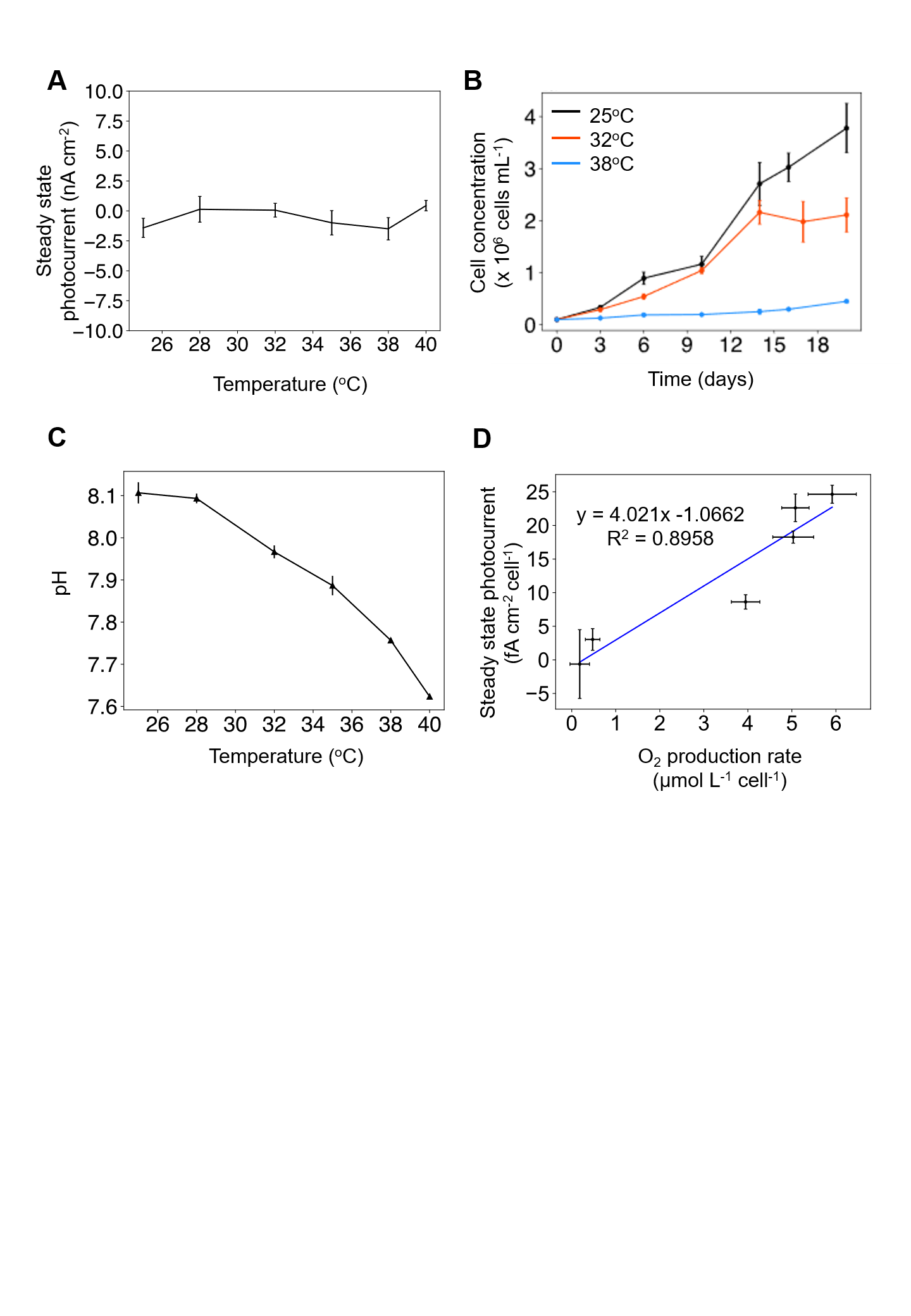
**

Fig. S6.

Response of *S. microadriaticum* cells to different temperatures.

(**A**) Steady state photocurrents at increasing temperatures without *S. microadriaticum* added. (**B**) Effect of temperature on the growth of *S. microadriaticum*. (**C**) Effect of temperature on the pH of f/2 medium without cells. (**D**) Linear regression analysis of steady state photocurrent versus oxygen production in changing temperatures. For B and D, data shown are averages of three biological replicates, error bars represent the standard error of the mean. Other data shown are averages of five (A) or three (C) technical replicates, error bars represent standard error of the mean.


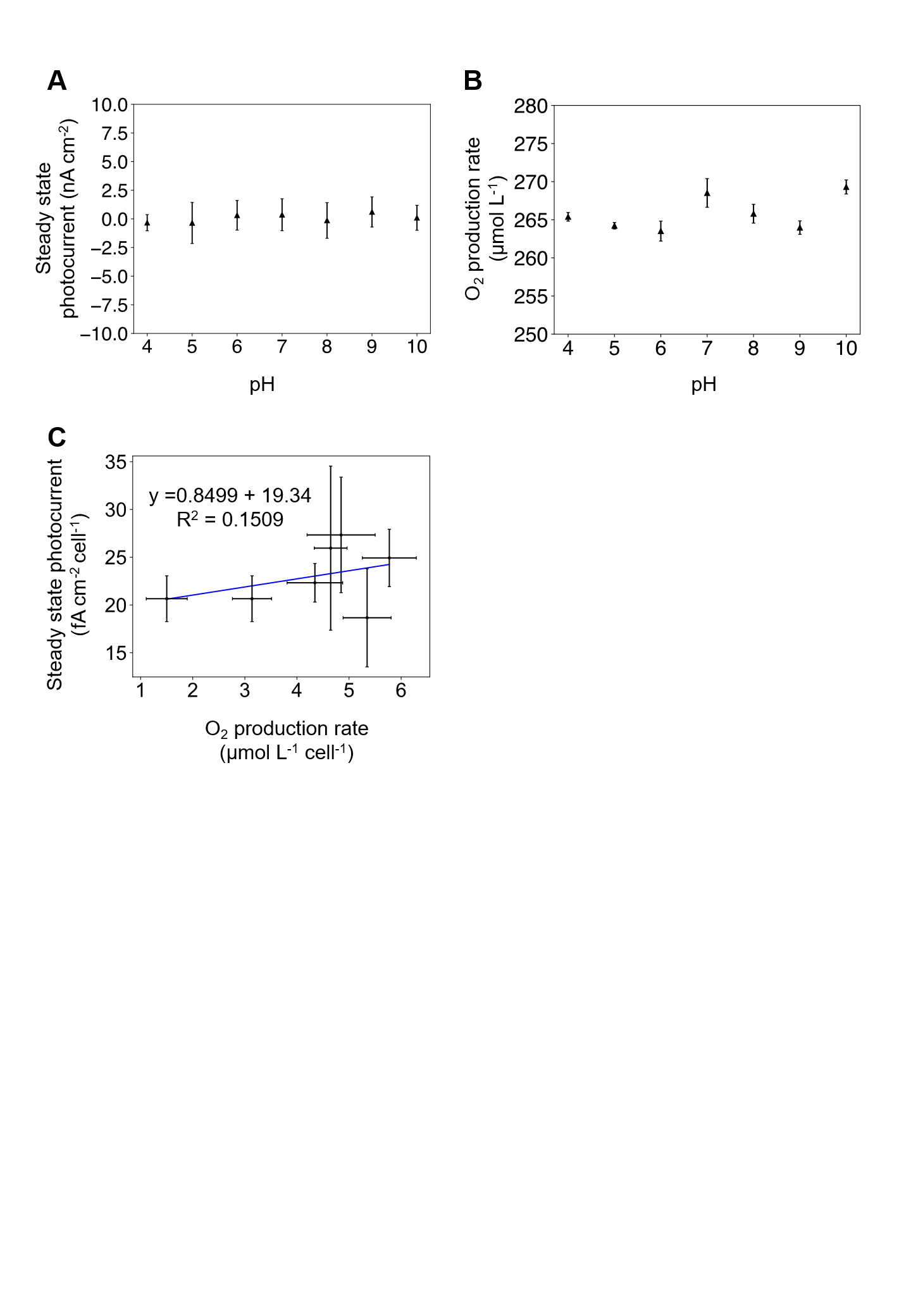


Fig. S7.

Response of *S. microadriaticum* cells to different pH levels.

(**A**) Steady state photocurrent at different pHs in f/2 without *S. microadriaticum*. (**B**) Oxygen concentration in f/2 medium at different adjusted pHs, without *S. microadriaticum*. All photocurrent measurements were obtained at 0.3 V vs SHE, with 680 nm light at 50 μmol photons m^-2^ s^-1^. (**C**) Linear regression analysis of steady state photocurrent versus oxygen production in changing temperatures. For C, data shown are averages of three biological replicates, error bars represent the standard error of the mean. Other data shown are averages of five technical replicates, error bars represent standard error of the mean.
